# More friends on SNS, more materialism? The moderating roles of self-esteem and social comparison orientation

**DOI:** 10.1371/journal.pone.0283723

**Published:** 2023-05-10

**Authors:** Chenhan Ruan, Zhihuang Lu, Huizhong Li, Wenhe Lin, Dan Li, Jingting Yuan

**Affiliations:** 1 School of Economics and Management, Fujian Agricultural and Forestry University, Fuzhou, China; 2 The Faculty of Psychology, Beijing Normal University, Beijing, China; 3 Guangzhou Automobile Group Co., Ltd. R & D Center, Guangzhou, China; 4 China Construction Bank, Beijing, China; Beijing University of Technology, CHINA

## Abstract

On social networking sites, users are continuously exposed to a variety of posts from the networked individuals. Such information may often influence recipients’ perceptions of what is important and goal pursuits such as materialism. Even though several studies have examined the negative consequences of using social networking sites, less attention has been paid to the role of friends’ number and its impact on people’s life goal pursuits. This study aimed to investigate the dark side of online friends and explored why and when more friends in social networking sites would promote materialism. Based on a sample of 264 WeChat users, study 1 discovered that friends’ number positively impacted materialism through extrinsic goal (i.e., wealth and status). Additionally, such association was moderated by social comparison orientation and self-esteem. Importantly, self-esteem buffers the detrimental effect of friends’ number on materialism while social comparison orientation increases it. Study 2 further tested the causal relationship and showed that friends’ number on SNS might become a signal to indicate materialism via an experiment. In conclusion, our findings add to the understanding of psychological processes regarding the dark side of online friends’ number and render suggestions for developing positive personal value.

## Introduction

Social networking sites (SNSs), such as Facebook, WhatsApp and WeChat, are regarded as a powerful tool for connecting and information sharing. For instance, active Facebook users reached 2.91 billion in the United States [1] and WeChat, the most widely used SNS in China connected more than 1.26 billion monthly active users [2]. On SNSs, users can freely present experiences, feelings and thoughts for self-presentation with many online friends [3,4]. In the meantime, they are also continuously exposed to updated posts from networked friends. Such information may subsequently shape their own value and life goal pursuits. Given that friends in SNSs exhibit different interaction modes and tie strength [5,6], it would be interesting to figure out how more friends in SNSs influence users’ values such as materialistic tendency.

Friends in SNSs refer to the connections based on SNSs that share profiles, information and maintain relationship [7,8]. Classical studies on relationship formation focus on the positive aspects of friends’ number. Since online friends serve as social capital [5,6], connections with friends in SNSs would bring trust, happiness [9], sense of belonging [10] and life satisfaction [11,12], especially for students facing cross-cultural adaption [13]. On the contrary, some research indicates that more friends may induce negative emotional outcomes such as SNSs’ fatigue [14–16], depression [17–19], fear of missing out [17] and loneliness [20].

Although recent studies started to focus on some materialistic behaviors by SNSs’ usage such as impulsive buying [12,21,22] and conspicuous consumption [23], it is still unclear whether and how friends’ number would drive the development of materialistic tendency. In our research, we argued that SNS friends’ number positively lead to materialism value, the belief in the importance to acquire money and possessions [24]. Specifically, information shared by SNSs’ friends comprises the details of possessions, success and some positively-skewed pictures of realities [25,26]. More friends, more exposure chances to the materialistic and idealized information, which prompts people to compare themselves with their online friends [3], and the upward comparison induces the desire for material and successful lives, i.e., materialism [23,27].

Therefore, this paper attempted to explore the impact of SNSs’ friends’ number on materialism by means of a survey of WeChat users and an experiment. The current research contributed to the literature in the following ways. First, prior studies discovered the positive outcomes of friends’ number such as trust and security [8]. We explored the dark side of SNSs’ friends and revealed that they were important source of social comparison that encouraged materialism. Besides, although recent literature started to examine the relationship between SNSs usage and some materialistic behaviors [27], it lacked to examine whether and how friends’ number would affect materialism. Thirdly, we identified specific personal characteristics that influence the relationship between friends’ number on materialism. Specifically, we claimed that social comparison orientation and self-esteem would help to predict who are more vulnerable to the negative psychological outcomes brought by friends’ number. Understanding why and when SNSs’ friends’ number lead to materialism is not only theoretically important, but also has practical implications for interpersonal relationship and value development in SNSs.

## Theoretical background and hypotheses

### The effect of number of SNSs’ friends on materialism

SNSs provide a convenient venue for social connection and information sharing among online friends [7]. Different from friends offline which require strong attachment or close connections, friends in SNSs refer to the weaker, online relationship concerning “the list of friends who establish relationship through SNSs, connected by sharing profiles, information and communication online” [7], even without face-to-face meeting.

Friends on social media have some unique properties compared to the traditional offline interactions. On the one hand, social media facilitate communications and interactions among networked friends [6]. More friends nowadays means more interactions and psychological support [8,9,11], which lead to positive outcomes including trust, happiness [9], sense of belonging and life satisfaction [11,12]. Kim and Lee found that the number of Facebook friends had a positive relationship with one’s subjective well-being [8]. On the other hand, users on social media establish relationships with weak ties and strong ties [6]. More friends in SNSs indicated more social capital [5,6], especially for ones in need of social support such as foreign students facing cross-cultural adaption [13]. Pang demonstrated that WeChat network size could positively influences bridging, bonding and social capital maintenance [12].

Besides, some studies explored the negative aspects of number of SNSs’ friends including unfavorable emotional consequences like SNSs’ fatigue [14–16], mental distress and loneliness [20]. For instance, Brandtzæg, Lüders, and Skjetne pointed out that users with many friends may be overwhelmed with the need to process too much information [14]. Pang explored the relationship between WeChat engagement on negative consequences like depression mood and fear of missing out [17]. Some recent findings extended from emotional to some materialistic behaviors by SNSs’ usage including impulsive buying [12,21,22] and conspicuous consumption [23]. So, it is interesting to figure out whether and how friends’ number would lead to material goal pursuits.

This paper argued that friends’ number would lead to high materialism. Materialism can be seen as an internal value concerning the extent to which they believe happiness comes from possessions and acquisitions are the central goal in life, and success in life is measured by material achievement [28]. Holt expressed a similar sentiment when he conceptualized materialism as “the consumption style that results when consumers perceive that value inheres in consumption objects rather than experiences and people” [29]. For one thing, the posted messages on SNSs usually comprise the details of people’s possessions [25] to express their identity [30]. More friends in SNSs increase the exposure chances to friends’ lives with details of their possessions [25], and continuously exposure to peers’ materialistic information would constitute a social influence that greatly change their personal value towards materialism [31,32]. For another, friends’ posted contents are mostly idealized and positively-skewed [26], which are more likely to induce upwards social comparison [33,34]. This would subsequently lead to negative self-evaluation [35] and fear of missing out in passive SNSs usage [17,36]. As a compensatory strategy towards the psychological threat, people would increase materialism [31,32,37], especially in the consumption domain. This is in line with previous finding that social comparison, especially among peers, contributed to people adopting materialistic values [22,38]. Although some research demonstrated the positive link between SNSs’ usage and materialism [27], there is no direct evidence to prove the relationship between SNSs friends’ number and materialism, Therefore, we proposed that:

**H1:** The more friends on SNSs, the greater materialism.

### The mediating role of extrinsic goal

According to self-determined theory [39], extrinsic goals are characterized as an outward orientation driven by making good impressions on others, including good physical looks, public image (fame), and financial achievements. This is in line with Kasser and Ryan that aspirations for financial success, social recognition and appealing appearance were extrinsic goals typically engaged in as means to some other ends [40].

From displaying self-images to one’s possessions, friends’ posts provide a variety of external cues to activate the external goals [41,42]. For example, observing luxury brands activates the social status motive, one typical type of external goals to gain reputation and social recognition [43]. Attention to the appealing selfies enhances the goal of physical attractiveness [40] and moreover, exposure to information of possessions and wonderful life increases the aspiration for financial success [44]. The external cue activates people’s extrinsic goal to win favor from others in a way they appreciated. It changes their perceptions about what contributes to happiness and success in life and increases the desire for acquisition and possession of material objects (materialism by definition) [28].

On the other hand, the majority of information posted by friends is idealized and positively-skewed [26]. Browsing information from superior peers generates the feeling of relative depravation that one has fewer possessions and lower abilities than others [45]. This perception would be augmented in SNSs where upward comparison is prevalent [17,46]. When consumers experience the psychological threat from lacking something their peers own, they would turn to other outward rewards for compensatory. As a result, they increase the extrinsic goals to buffer the stressors in an aversive situation [47,48]. So, people with more friends exhibit higher extrinsic goals, stressing greater importance on extrinsic rewards like wealth and material possessions, which finally contributes to materialistic tendency [28].

**H2:** The effect of number of SNSs’ friends on materialism is mediated by the emphasis on extrinsic goal.

### The moderating role of social comparison orientation

We further explored social comparison orientation and self-esteem as two important characteristics that may influence the detrimental effects of friends’ number on materialism. Social comparison orientation refers to individual differences in people’s tendency to compare themselves with others [49]. Individuals with high social comparison orientation base their self-evaluation on how others are doing, while individuals with low social comparison orientation focus more on themselves and are more resistant to outside influences [49]. Although it is a natural tendency for people to compare themselves with others in SNSs [35,46], people high in social comparison orientation are more susceptible to others’ positively-biased posts and are more likely to adopt values such as materialism.

This is because first, individuals with high social comparison orientation have high social comparison frequency in SNSs [35,50–53]. They rely on outside information to reduce uncertainty about the self [49], and are more sensitive and attentive to crucial materialistic information shared by friends [37]. Such materialistic information further enhances the endorsement of materialism value [54]. Similarly, Vogel et al. proved that higher social comparison orientation could predict more SNS usage and caused detrimental effects on Facebook [51]. Second, people with high social comparison orientation hold uncertain and unstable self-perception [49]. Continuously exposure to positively biased information induces the psychological threat such as identity threat, Facebook-related attachment anxiety [3], as well as negative perceptions of one’s own attractiveness and social competence [55]. To compensate for the psychological threat induced by friends’ posts, they would increase materialism to recover mental health [31,32,56,57]. For example, Ho and Ito found that young adults high in social comparison orientation experienced overspending with more engagement in SNSs [58]. On the contrary, Individuals with low social comparison orientation report less frequent social networking sites usage and upward social comparison [51]. They focus more on themselves, show stable self-concepts, and are more resistant to external influence [49]. Thus, the impact of friends’ number on materialism would be weakened for them.

Therefore, we hypothesized the following:

**H3:** Social comparison orientation moderates the effect of SNSs’ friends’ number on materialism: for consumers with high social comparison orientation, the number of friends is positively associated with the materialism; for consumers with low social comparison orientation, the positive impact of friends’ number on materialism is attenuated.

In addition, people high in social comparison orientation are chronically sensitive to what others think about them [49]. Greater concern about being socially evaluated on Facebook is manifested by excessive reassurance seeking, need for approval and attention seeking [3]. The pursuit of outward recognition and social approval for a better sense of self promotes the extrinsic goals [40,59], which subsequently leads to materialism [32]. Therefore, for people high in social comparison orientation, more SNSs’ friends, more exposure to others’ positive life, more tendency for them to promote extrinsic goals and materialistic behaviors like luxury consumption [44,56]. However, for people with low levels of social comparison orientation, they present clear self-identity [3], focus more on themselves [49], and thus are less influenced by friends’ number in SNSs. Thus, we proposed that:

**H4:** Social comparison orientation moderates the effect of SNSs friends’ number on materialism via extrinsic goal: for consumers with high social comparison orientation, the number of friends is positively associated with extrinsic goal, which subsequently results in high materialism; for consumers with low social comparison orientation, the positive impact of friends’ number on materialism via extrinsic goal is attenuated.

### The moderating role of self-esteem

Self-esteem constitutes a perception or evaluation of self-worthiness [60]; that is, the extent to which an individual views the self as worthwhile and competent [61]. Individuals with high self-esteem are generally more optimistic and confident, and less likely to be influenced by environment [61,62]. On the contrary, those with low self-esteem have low confidence about self. As a result, they are more likely to seek external information such as social comparison for clear understanding about self [46].

In the context of SNSs, for people with high self-esteem, they are less likely to be influenced by friends’ number since they rely less on external information for self-evaluations [46]. It serves as a buffer against social comparison information posted by many friends that may be harmful to the overall well-being [63]. Similarly, prior studies reveal that self-esteem is negatively related with SNS usage and upward social comparison [35], which subsequently contributes to positive mental and behavioral outcomes [46,51]. As a result, high self-esteem protects users against detrimental effects of friends’ number on materialism. However, for individuals with low self-esteem, they exhibit higher need of comparison information on Facebook [35,46]. They are more susceptible to social comparison posts [35,64] and adapt materialistic value as a result of emotional compensation [65].

**H5:** Self-esteem moderates the effect of SNSs friends’ number on materialism: for consumers with low self-esteem, the number of friends is positively associated with materialism; for consumers with high self-esteem, the positive impact of friends’ number on materialism is attenuated.

In addition, according to the previous literature, people with high self-esteem tend to establish their self-worth in the internal contingencies, while those with low self-esteem establish their self-worth in the external contingencies such as appearance and approval from others [66]. On the one hand, a flow of materialistic information in SNSs constitutes a social influence to signal that seeking wealth and status are universal ways to gain social approval [31,32]. Because people with low self-esteem depend more on extrinsic social approval for self-recognition, more exposure to posts from friends enhances their extrinsic goals to seek social recognition [59], and thus results in materialism [32]. On the other hand, the activation of extrinsic goals motivates the materialistic behavior intended to construct and maintain self-identity [48]. People with low self-esteem tend to rely more on material goods when their extrinsic goals are promoted [59]. However, individuals with high self-esteem could demonstrate their personal worth and status by emphasizing their abilities or strengths, and they do not need to prove themselves through material wealth [28]. Therefore, we hypothesized that:

**H6:** Self-esteem moderates the mediating route of number of friends on materialism via extrinsic goal: for consumers with low self-esteem, the number of friends is positively associated with extrinsic goal, which subsequently results in high materialism; for consumers with high self-esteem, the positive impact of friends’ number on materialism via extrinsic goal is attenuated.

**Fig 1:**
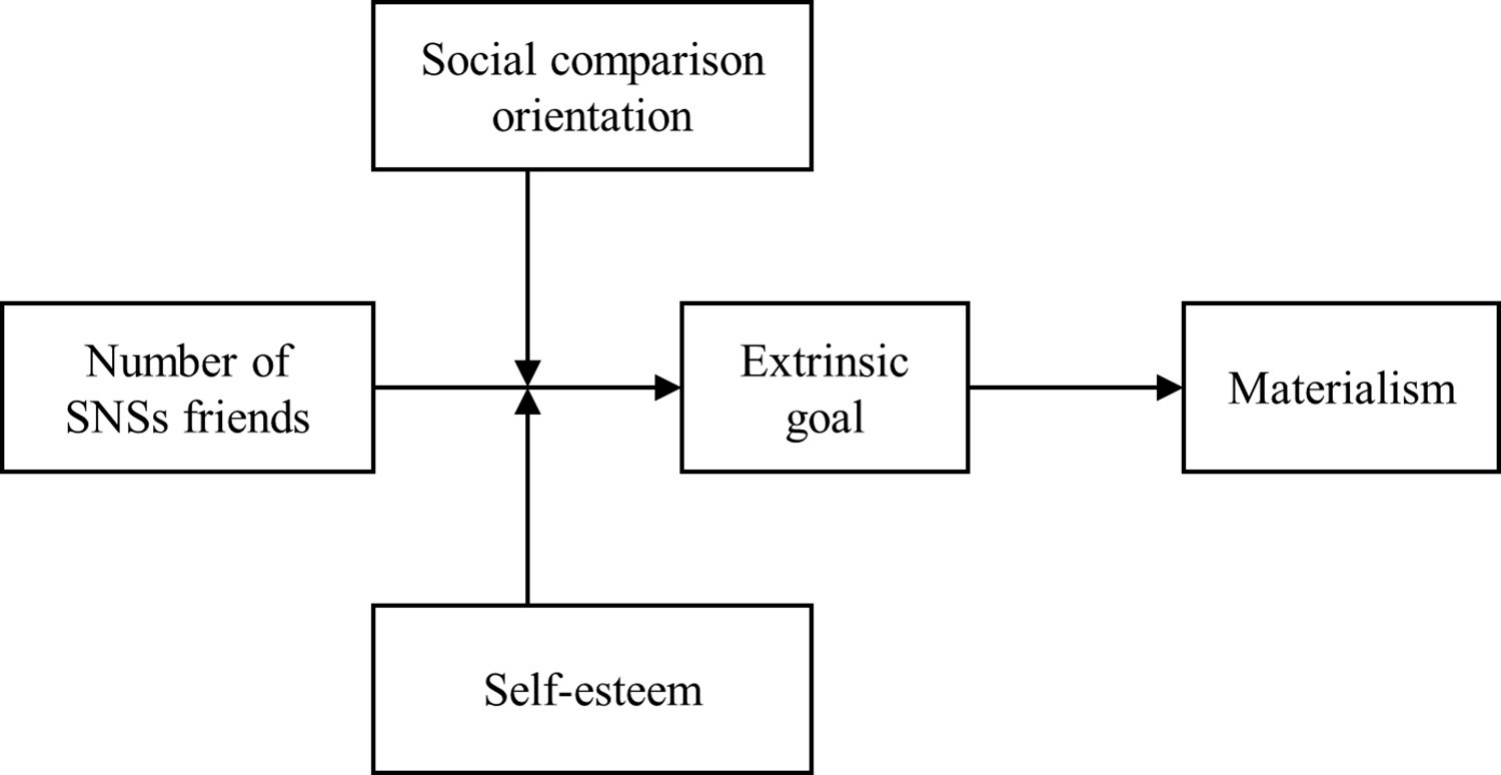
Theoretical framework.

Based on the above theoretical foundations, we derived the theoretical framework for this research in Fig 1.

## Study 1

### Participants and procedure

We chose *WeChat* as the target type of SNS in the current research because WeChat is the most representative SNS among Chinese users, and has been the main data source for studies about SNSs in China [10,11,64]. The study was approved by the Institutional Review Board (IRB) of Fujian Agricultural and Forestry University. Undergraduate students that registered in the participant pool in China were emailed with a recruitment message by WeChat, a widely used social media platform in China. They were directed to an online survey website by a hyperlink in October 2021. Each participant was informed of the online formal consent before they enrolled in the present study. The first page of the questionnaire addresses the purpose of this study, the authenticity, independence, confidentiality and integrity of all answers. After reading the online formal consent and instructions, they were informed to finish the measures, involving number of WeChat friends, materialism, external goal, and some demographic variables including gender and age.

Out of the 280 registrants who received the e-mail, 264 students completed the survey in exchange for $4 (response rate: 94.28%; female: 71.3%; male: 28.7%), age ranges from 18 to 27 years old (M_age_ = 20.7, SD = 1.90). On average, participants had about 225 friends on their WeChat accounts.

### Measures

All the variables were assessed using measures that had been frequently applied and validated in existing research. We followed the translation and back-translation procedure to validate the translation quality. We pretested it among a small set of participants to make sure all items were easy to understand and without ambiguity.

*Number of SNS’s friends* was evaluated by asking participants how many friends in total they had in their WeChat on a 9-point scale (0 = 10 or less, 1 = 11–50, 2 = 51–100, 3 = 101–150, 4 = 151–200, 5 = 201–250, 6 = 251–300, 7 = 301–400, 8 = more than 400) [9].

*Materialism* was measured with 9-item Material Values Scale developed by Richins and Dawson [28]. Specifically, Material Values Scale consists of three dimensions: the centrality of acquisition to a person’s life (e.g., “Buying things gives me a lot of pleasure”), the believes that acquisition provides happiness (“I’d be happier if I could afford to buy more things”) and signifies success (e.g., “I admire people who own expensive homes, cars, and clothes.”). The Cronbach’s *α* of this measurement was 0.66.

*Extrinsic goal* was assessed using the aspiration index developed by Kasser and Ryan [40,67]. Specifically, participants rated the importance of different aspirations on 7-point scales (1 = strongly disagree, 7 = strongly agree). We measured extrinsic goal with 3 kinds of external aspirations including “You will be wealthy and materially successful (financial success)”, “You will be famous, well-known and admired (social recognition)” and “You will look attractive in terms of body, clothing and fashion (appealing appearance)” (*α* = 0.78).

*Social Comparison Orientation* was measured according to the Iowa-Netherlands Comparison Orientation Scale developed by Gibbons and Buunk [49], which consisted of 11 items on a 7-point Likert-type scale (1 = strongly disagree, 7 = strongly agree). Sample questions included “I always pay a lot attention to how I do things compared with how others do things”, “If I want to find out how well I have done something, I compare what I have done with how others have done”, “I often compare how I am doing socially (e.g., social skills, popularity) with other people” (*α* = 0.85).

*Self-esteem* was evaluated with a 10-item scale developed by Rosenberg Self-esteem Scale [61]. Participants responded sample items including “I take a positive attitude towards myself”, “One the whole, I am satisfied with myself”, “I feel that I have a number of good qualities” (*α* = 0.89).

### Data analysis

We first conducted a path analysis model with materialism as outcome, SNS friends’ number as the predictor in Mplus 8.0. Next, we included mediator and tested the indirect effect through extrinsic goal by a Monte Carlo simulation approach [68]. Additionally, we added social comparison orientation and self-esteem as moderators in the model, and conducted simple slope analyses to examine moderation effects. The Monte Carlo simulation approach was also used to estimate the moderated mediation effects of social comparison orientation and self-esteem.

*Common Method Bias*: As the data was collected using a self-report questionnaire and all variables are perceptual measures derived from respondents, common method bias (CMB) may be a concern. Both procedural and statistical remedies were used to control CMB as suggested by Podsakoff et al. [69]. Procedurally, participants were assured that they were anonymous and that there were no correct or incorrect answers. The statistical remedies included Harman’s one-factor test to assess the presence of CMB [69]. All the items of the study were entered into a principal component analysis. The results showed that the first factor contributed 18.26% of the variance and that there was no single factor in the factor structure. Therefore, CMB was unlikely to affect the results [69].

### Results

#### Descriptive statistics

The descriptive information of all variables was presented in Table 1, including means, standard deviations, and Pearson correlations (see Table 1).

**Table 1:** Descriptive statistics and correlations among variables.

Variables	*M*	*SD*	1	2	3	4	5
The number of SNS’s friends	6.18	2.17	--				
Materialism	3.25	0.52	0.10	--			
Extrinsic goal	4.99	1.04	0.21**	0.57**	--		
Self-esteem	5.06	0.96	0.15*	-0.11	0.01	--	
Social comparison orientation	3.16	0.67	0.26**	0.23**	0.16**	-0.05	--

Note: N = 264.

* *p* < 0.05.

** *p* < 0.01.

### Hypotheses testing

#### The effect of number of SNSs friends on materialism

A regression model with materialism as the dependent variable, the number of friends as the independent variable was performed. The results showed a marginally significant effect of friends’ number on materialism (*β* = 0.10, t = 1.69, *p* = 0.09), which partially supported Hypothesis 1.

#### The mediating role of extrinsic goal

To test the mediating role of extrinsic goal for the relationship between friends’ number and materialism, we performed a series of regressions [70]. Results showed a positive relationship between friends’ number on extrinsic goal (*β* = 0.21, t = 3.43, *p* < 0.05). Besides, materialism was positively associated with extrinsic goal (*β* = 0.57, t = 11.09, *p* < 0.001). When we controlled for extrinsic goal, the effect of friends’ number on materialism wasn’t significant anymore (*β* = -0.01, t = -0.27, *p* = 0.79), which suggested a full mediation effect.

To further verify the mediation effect, we conducted a mediation analysis using bootstrapping (Model 4, based on 5,000 samples [71]). Results demonstrated that extrinsic goal mediated the effect of friends’ number on materialism (Indirect effect = 0.03, *s*.*e*. = 0.01, 95% CI = [0.01, 0.05]). Hence, hypothesis 2 was supported.

#### The moderating role of social comparison orientation

Next, to test the moderating role of social comparison orientation on the relationship between friends’ number and materialism, we conducted a moderated mediation model. The results indicated a significant main effect of friends’ number (*β* = -0.64, t = -2.17, *p* < 0.05), a non-significant main effect of social comparison orientation (*β* = -0.22, t = -1.16, *p* = 0.25), and a significant interaction effect (*β* = 0.92, t = 2.39, *p* < 0.05), which meant that social comparison orientation served as a moderator to boost the positive impact of friends’ number on materialism. This moderating effect was further verified with bootstrapping (model 1, based on 5,000 samples [71] (Effect = 0.05, *s*.*e*. = 0.02, *p* < 0.05, 95% CI = [0.01,0.10]).

**Fig 2:**
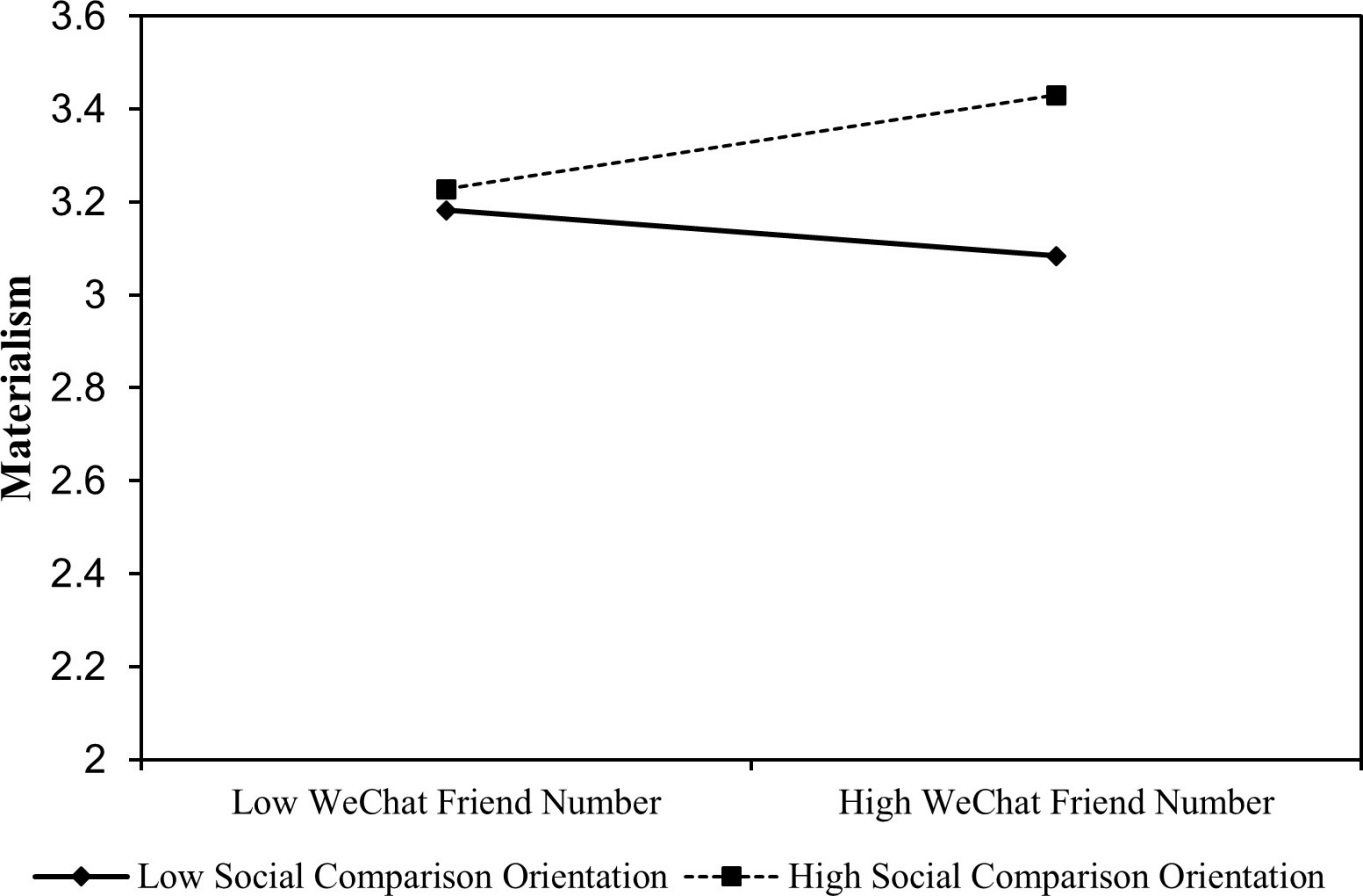
Simple slope analysis for social comparison orientation.

Furthermore, A simple slope analysis showed that the effects of friends’ number on materialism was stronger for people with high levels of social comparison orientation (Effect = 0.05, *s*.*e*. = 0.02, *p* < 0.05, 95% CI = [0.01,0.09]), compared to people with low levels of social comparison orientation (Effect = -0.02, *s*.*e*. = 0.02, *p* = 0.27, 95% CI = [-0.06,0.02]; Fig 2), which supported hypothesis 3.

**Fig 3:**
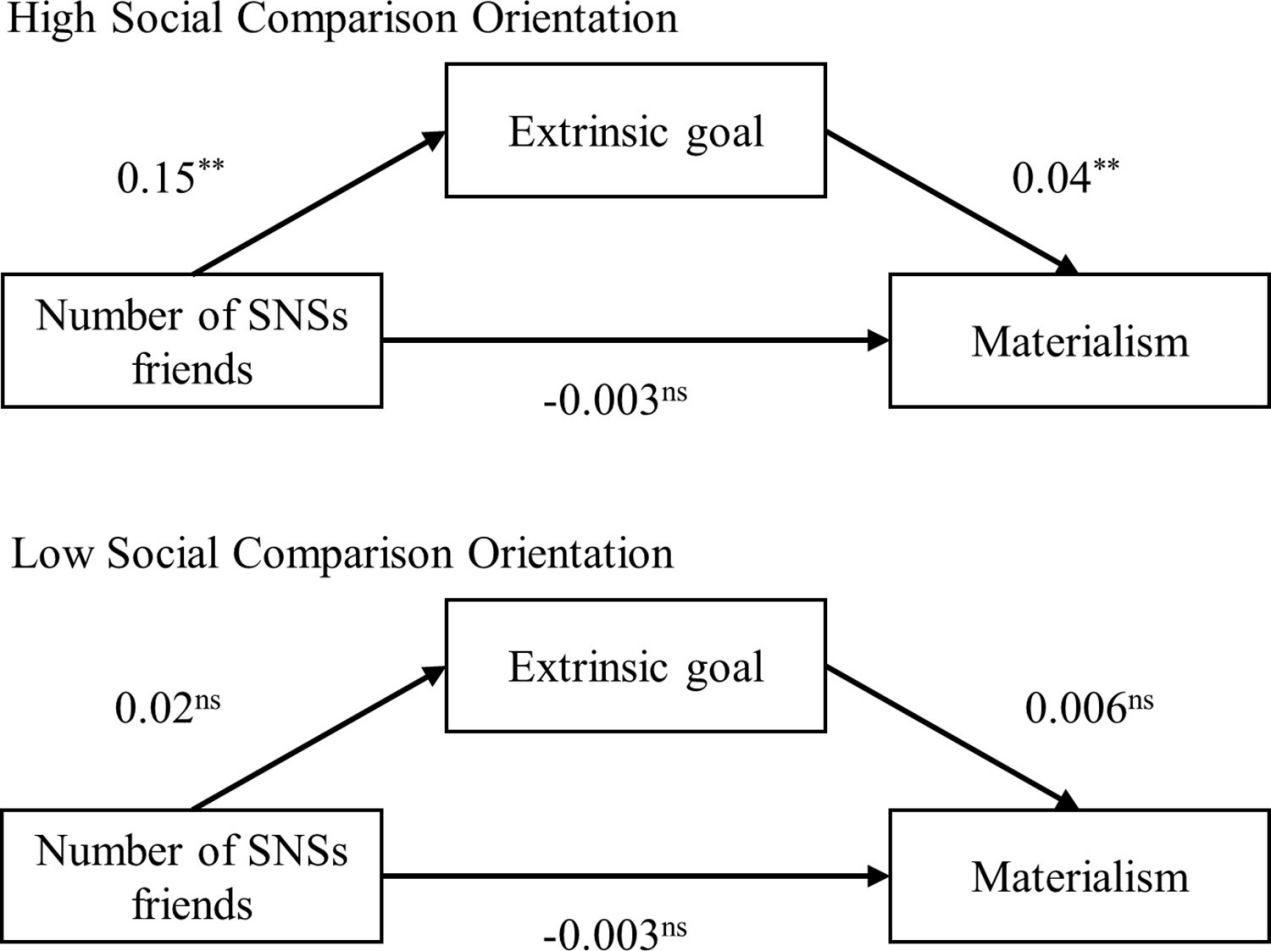
Moderated mediation effect for social comparison orientation.

In addition, there was a significant moderated mediation effect of social comparison orientation on the indirect route from friends’ number on materialism via extrinsic goal (Effect = 0.03, *s*.*e*. = 0.01, 95% CI = [0.01, 0.05]). Specifically, for people with high social comparison orientation, extrinsic goal significantly mediated the relationship between friends’ number and materialism (Effect = -0.04, *s*.*e*. = 0.01, 95% CI = [0.19, 0.07]; Fig 3). Conversely, for people with low social comparison orientation, the mediating effect of extrinsic goal between friends’ number and materialism was non-significant (Effect = 0.01, *s*.*e*. = 0.01, 95% CI = [-0.02, 0.29]; Fig 3). The results suggested that for people high in social comparison orientation, more friends in SNS prompted them to seek extrinsic goal such as status and wealth, which therefore enhanced their materialism. In contrast, when people perceived low levels of social comparison orientation, interactions with SNSs friends would not significantly drive them to pursuit the extrinsic goal and change their materialistic tendency.

#### The moderating role of self-esteem

Similarly, we performed a moderated mediation model with friends’ number as independent variable, materialism as dependent variable, extrinsic goal as mediator, and self-esteem as the moderator variable. In support of H4, there was a significant main effect of friends’ number (*β* = 0.88, t = 2.77, *p* < 0.05), a non-significant main effect of self-esteem (*β* = 0.27, t = 1.53, *p* = 0.13), and a significant interaction effect (*β* = -0.92, t = -2.43, *p* < 0.05). The results showed that self-esteem weakened the effect of WeChat friends on materialism (*β* = -0.04, *s*.*e*. = 0.02, *p* < 0.05, 95% CI = [-0.07, -0.01]).

**Fig 4:**
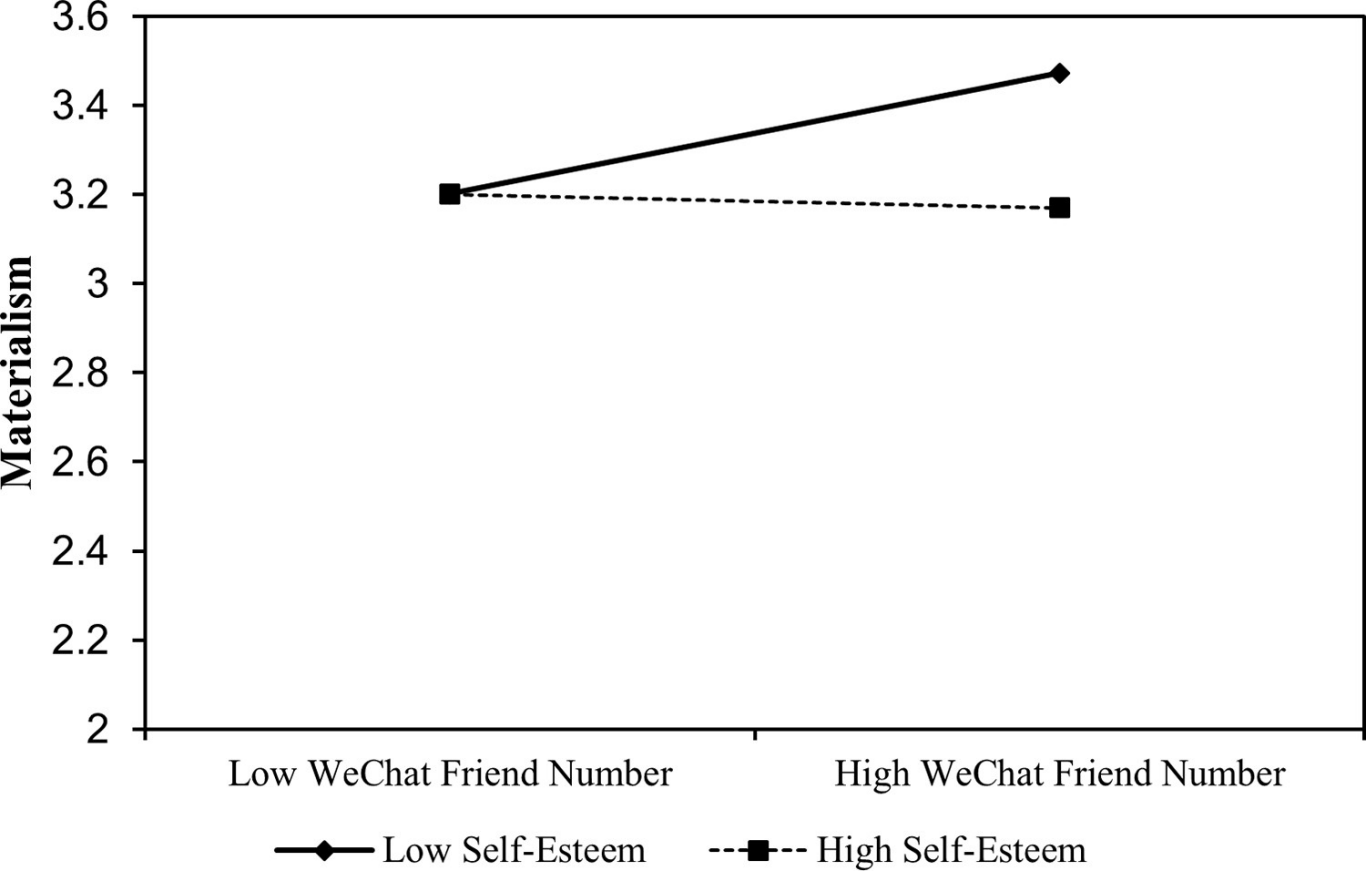
Simple slope analysis for self-esteem.

A simple slope analysis confirmed that the effects of friends’ number on materialism was stronger for people with low levels of self-esteem (*β* = 0.06, *s*.*e*. = 0.02, p < 0.05, 95% CI = [0.02,0.10]), compared to people with high levels of self-esteem (*β* = -0.01, *s*.*e*. = 0.02, *p* = 0.74, 95% CI = [-0.05,0.03]; Fig 4).

**Fig 5:**
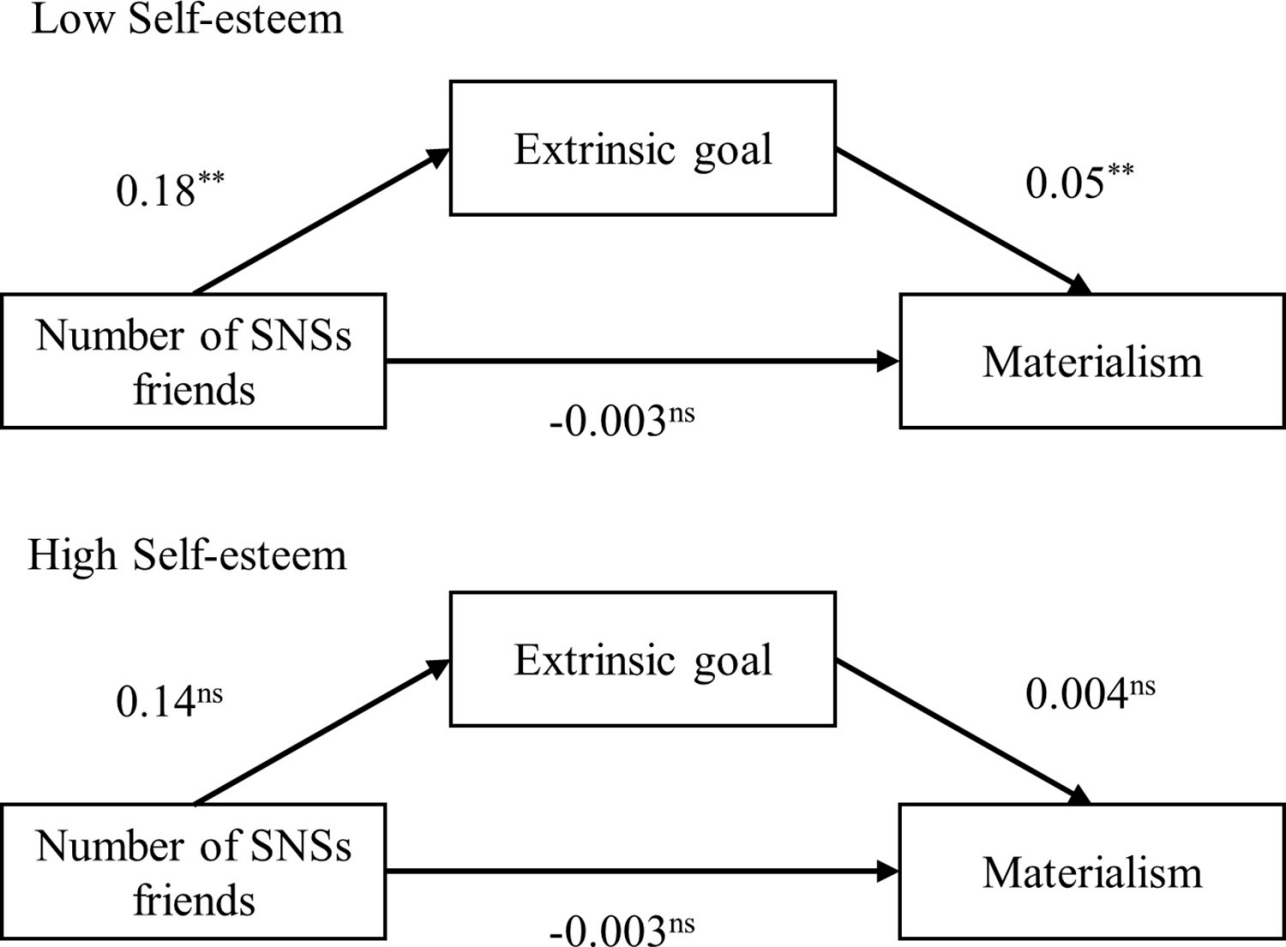
Moderated mediation effect for self-esteem.

As hypothesized, there was significant moderated mediation effect of self-esteem on the indirect route from friends’ number on materialism via extrinsic goal (Effect = -0.02, *s*.*e*. = 0.01, 95% CI = [-0.04, -0.01]). Specifically, for people with low levels of self-esteem, extrinsic goal significantly mediated the relationship between friends’ number and materialism (Effect = 0.05, *s*.*e*. = 0.01, 95% CI = [0.02, 0.08]; Fig 5). Conversely, for people with high levels of self-esteem, the mediating effect of extrinsic goal between friends’ number and materialism was non-significant (Effect = 0.004, *s*.*e*. = 0.01, 95% CI = [-0.02, 0.03]; Fig 5). The findings demonstrated that people with low levels of self-esteem were more vulnerable to the negative consequences by posts from WeChat friends, due to the fact of their increase motivation for extrinsic goal.

## Study 2

The objective of this study is to use a controlled experimental setting to dissect the relationship between friends’ number on materialism on a general SNS platform. We employed a 2 (number of SNS’s friends: high vs low) factorial between-subjects experimental design. Specifically, we designed the experiment from the recipients’ perspective since as users of the SNS, they know how they were affected by friends, and thus to ensure a positive inferences of materialism in terms of friends’ number.

### Participants and procedure

The study was approved by the Institutional Review Board (IRB) of Fujian Agricultural and Forestry University. Participants finished the survey via an online survey platform in China (http://www.credamo.com). 160 participants completed the survey in exchange for 2 CNY (female: 64.4%; male: 35.6%). 88.8% of the participants were between the ages of 21 and 40.

Each participant was informed of the online formal consent before they enrolled in the present study. After confirming their online consent, participants were randomly assigned to one of two conditions: high friends’ number or low friends’ number. They read an imaginary blogger’s personal profile called Sunnie from a SNS platform. We directly manipulated friends’ number by displaying different friends’ numbers on the profile, where in the high number condition he had 2500 friends and in the low number condition he had only 25 friends. As in study 1, participants All were then asked to rate Sunnie on materialism (α = 0.96, i.e., to what extent they think “Sunnie admires people who own expensive homes, cars, and clothes”), extrinsic goal (α = 0.81, i.e., to what extent they think “Sunnie aspires for the importance of being wealthy and materially successful”). Next, participants were asked to complete the manipulation checks with the following two questions, “to what extent do you think Sunnie’s friends’ number on that SNS is (1 = very little, 7 = very much)”, “to what extent do you think Sunnie’s friends’ size on that SNS is (1 = very small, 7 = very large)” (α = 0.94). Finally, they provided their demographic information including gender and age.

### Results

The analysis of the manipulation check confirmed that participants assigned to the high number condition perceived that the blogger had more friends (M = 5.51, SD = 1.00) than those assigned to the low number condition (M = 2.68, SD = 1.34; t(158) = -15.15, p<0.001). Thus, the manipulation of the number of SNS’s friends was successful. Besides, compared to participants in low friends’ number condition, participants who saw a profile with high friends’ number tended to perceive the blogger as more materialism (M_high_ = 4.69, SD = 1.57; M_low_ = 4.10, SD = 1.66; t(158) = -2.31, p < 0.05). This suggested that friends’ number exerts a significant influence on inferences of materialism. Participants thought Sunnie had higher materialism when he had more friends on SNS, which supported the positive relationship between friends’ number and materialism in H1.

#### The mediating role of extrinsic goal

To test the mediating role of extrinsic goal for the relationship between friends’ number and materialism, we performed a multiple-step mediation analysis [70]. As predicted, we found a positive relationship between friends’ number on extrinsic goal (*β* = 0.39, t = 5.27, *p* < 0.001). Besides, materialism was positively associated with extrinsic goal (*β* = 0.80, t = 16.64, *p* < 0.001). When we controlled for extrinsic goal, friends’ number was no longer a positive influence on materialism (*β* = -0.15, t = -2.91, *p* < 0.01), which suggested a full mediation effect.

To further verify the mediation effect, we conducted a mediation analysis using bootstrapping (Model 4, based on 5,000 samples [71]. Results revealed a significant indirect effect for the mediation role of extrinsic goal (Indirect effect = 0.85, *s*.*e*. = 0.20, 95% CI = [0.47, 1.26]). The study demonstrated that more friends on SNS led to higher inferences in terms of poster’s extrinsic goal pursuit, which in turn enhanced the extent to which this blogger is perceived as materialism. This could also suggested that for users on SNS, friends’ number serve as a signal of materialism since exposing to more positively-skewed information posted by friends enhanced the extrinsic goal for the users, ultimately leading to high materialism. Hence, hypothesis 2 was supported.

## Discussion

With the development of social networking sites, social news from strong and weak ties has been made pervasive. Continuously exposure to posts shared by friends not only influences some affective attributes, but also changes our goal pursuits in life. Prior work has provided valuable investigation on this issue. For example, they proved that friends as social capital contributed to life satisfaction [10–12]. More friends nowadays suggest more interactions and psychological support [8,9,11]. Recent findings also revealed that Facebook usage may hurt mental health [14–17]. Pang systematically probed the negative psychosocial consequences of WeChat adoption, where passive WeChat use brings depression mood and fear of missing out [17]. In this paper, we aimed to explore the dark side of friends’ number on people’s life goal pursuits, and we are interested in whether more friends in SNSs can predict materialistic value tendency. Based on two studies, we showed that more friends in SNSs led to higher materialism. Besides, this relationship is driven by the enhanced desire for extrinsic goal. More importantly, we demonstrated that self-esteem and social comparison orientation served as moderators. Specifically, social comparison promotes the impact of friends’ number on materialism whereas self-esteem reduces the association between friends’ number on materialism. Study 2 also suggested that friends’ number was related to positive inference of bloggers’ materialism.

### Theoretical contributions

Our findings contribute to the literature in several ways. First, our research contributes to the research on online friends. Because friends online exhibited different interaction modes compared to traditional offline interactions [5,6], friends’ number in SNSs would bring different psychological benefits as well as challenges. Previous literature mainly regarded friends on SNSs as a form of social capital and advocated their positive sides in boosting well-being [7,11,14,15]. Although recent work has demonstrated negative affective impacts associated with SNSs usage [17–19], what remained unclear was whether and how the friends’ number may affect consumers’ life goals like materialism. We thus felt that this issue was worthy of investigation because material success is the central life goal among consumers and have substantial impacts on society [24]. Therefore, understanding how individual materialism was affected by their social size offers valuable insights for both business and public policy making.

Second, although some recent studies have suggested that SNSs’ usage has had a positive impact on some materialistic behaviors like impulsive buying [12,21,22] and conspicuous consumption [23], the psychological mechanism underlying this relationship was unclear. In this study, we integrated consumer extrinsic goal into the link between friends’ number to materialism. Given the fact that continuously exposure to the positively biased information including possessions, status and wealth shared by friends in SNSs would change people’s perception of what is more important in life [3]. The perspective of changes in people’s inner value system about goal pursuits provides a novel and augments understanding of the psychological processes that lead to materialism in SNSs.

Last, our results enhance comprehension of some individual traits including self-esteem and social comparison orientation that may influence the relationship between friends’ number on materialism. Specifically, consumers with lower levels of self-esteem and higher levels of social comparison orientation were more susceptible to SNSs friends’ influence. Our findings suggested that enhancing self-esteem or reducing social comparison orientation may be an approach to buffer the negative outcome of friends’ number on materialism and foster positive value in SNSs.

### Practical implications

Our findings offer valuable insights to augment understanding on the change of people’s goal pursuits in SNSs and provide managerial implications for business and marketing in SNSs. First, our results show that friends in SNSs not only offer informational and social support, but also influence our materialistic tendency. Such negative outcome might exert impacts not only on subjective well-being [40,67], but also the value orientation of the entire society. Therefore, SNSs marketers are encouraged to foster good environment for information sharing, improve the design of their marketing appeals to correctly guide consumers’ preference for online relationship size.

In addition, the findings suggested that more friends in SNSs may not always be a good thing. Consumers need to keep in mind that it is better to seek friends’ quality rather than friends’ quantity on SNSs. So, users should realize that they are affected by many positively skewed realities shared by their friends, especially when they have high level of social comparison orientation and low level of self-esteem.

Moreover, users should also pay attention to the contents they share. Sharing things like self-growth and love in impression management in SNSs would be more beneficial for the interpersonal relationships, compared to simply sharing material possessions, wealth and status. Besides, passing information such as social recognition and social connections to reduce social comparison orientation and boost self-esteem would be helpful for the supportive interactions among SNSs friends.

### Limitations and future research directions

There are some limitations of this study that are suggestive of future research. First, we mainly study one social networking platform WeChat in China, and whether our findings can be generalized to other platforms is unknown. In fact, WeChat as a widely used SNS in China has more than 1.26 billion monthly active users [2]. It shares similar functions as other SNSs like Facebook, including establishing connections, sending messages, and posting life events. It has been the main data source for many studies about SNSs in China [64]. For example, Pang used the data collected from WeChat to investigate the effects of different functions used by WeChat on university students’ online self-expression, social support and sense of belonging [10]. However, other platforms like TikTok or Instagram have different information displaying functions, which may affect the contents they exposed as well as the interactions among friends. Besides, for some short video platforms like TikTok, even with a small friends’ size, users can access a variety of short videos and live streams from strangers that may incorporate things like materialism and social comparison. So, in this context, the effect of friends’ number on materialism may change. Although we designed an imaginary SNS platform in Study 2, our findings need further validation with samples from various platforms and contexts.

Second, this study recruited 264 undergraduate participants in China, so most participants were young Chinese students mainly from a single university. Prior studies may consider student sample to study topics about SNSs [10–12,21]. For example, Tran et al. collected student sample in three universities in the south of Vietnam [21]. However, considering the wide usage of WeChat, it might limit the generalization to other populations such as older persons and adolescents. Also, samples from other cultures like United States may have a racially and ethnically diverse population. They may display different values towards information received from friends. Further studies are needed to repeat our results in other populations and cultures.

Third, although we discussed the moderation roles of self-esteem and social comparison orientation, our research could explore additional moderators associated with platform characteristics. One important boundary condition is the tie strength. Tie strength has been shown to influence the communication effects. For example, Valenzuela et al. found that Facebook use was more effective for strong-tie network [72]. We believed that the effect of relationship between friends’ number on materialism was more prominent for strong-tie network. According to Tesser’s, the upward social comparison threat on personal evaluation was stronger when the compared target was psychologically close than when the target was distant (similar/friend vs. different/stranger) [73]. However, in the weak-tie condition, far psychological distance mitigates the negative effect of upward social comparisons and thus friends may not exert significant impacts with the information they shared [74]. Besides, another important boundary condition is the platform position. If users use the platform to display their true life and gain social support [72], they may display fewer material purchases for high authenticity perception [75]. However, if platform is used to seek influence and social recognition by display novel things, users may tend to post some positively skewed pictures of reality for impression management and social appraisals [25,26]. Therefore, the impact of friends’ number on materialism may be enhanced. Also, future studies could systematically explore some individual characteristics and conditions which may influence to what extent users would post their lives or watch the posts to enrich the understanding of the dark side effects of the number of friends on SNS.

In addition, this was a study based on self-reported measures. Although we adopted mature scales to measure key variables, future research can incorporate more objective measures like google search index, content analyses or big data [18,19] to replicate our effect by looking at a larger number of SNSs users with a broader time interval. On the one hand, we could apply some objective measures and field data on friends’ number, interactions, social network structure, and indicators for materialism. On the other hand, it would be interesting to see how people change their attitudes when their friends increase in SNSs. Will extrinsic goal seeking generate any positive or negative responses among the networked others in the long run? How might people adjust their materialistic goal pursuit overtime? These are interesting questions that need further investigations over a longer time span.

## Supporting information

S1 Data (XLSX)
